# New Appliances and Things Medical

**Published:** 1894-09-01

**Authors:** 


### NEW APPLIANCES AND THINGS MEDICAL.

[All preparations, appliances, novelties, &c., of which a notice is desired, should be sent for the Editor, to care of The Manager 428, Strand, London, W.C.]

### SPECIALITIES FOR INVALIDS.

(Brand and Co., 11, Little Stanhope Street, Mayfair, W.)

At the invitation of the above firm, we paid a visit of inspection to their manufactory in the South Lambeth Road, S.W. Although as a general rule the management find it necessary to exercise a discriminating reticence as to their exact methods of preparation and manufacture, the senior partner, who conducted us from end to end of the vast buildings, put himself entirely at our disposal, and so far from making a secret of his methods, explained them fully and in detail; and in the case of a considerable number of the preparations of their well-known house, we had full opportunities of seeing every stage in the manufacture, from the cutting up of the meat to the soldering down of the tin or the corking of the bottles. We first inspected the large receiving room, where the meat is dissected from the bones, and subjected to the initial processes in the preparation of beef essences and beef teas. We were much struck by the quality of the meat, and with the cleanliness and freedom from unpleasant odours. Most people would imagine that bones, gristle, and fat would constitute a very good proportion of the total media from which such beef teas are manufactured. As a matter of fact the meat is most carefully freed from these less nutritious structures, and only a few bones are added to prevent scorching or burning of the meat. Great care is exercised in the tinning of such goods as are preserved by this method. All the joints of the cans are on the outside, so that none of the solder or soldered surface can come in contact with the contents, and in the majority of cases the final soldering down of the lid is completed with resin and not with metal, so that the most timid and most suspicious need have no fear of poisoning from this source in any of Messrs. Brand's goods. There is so much monotony in the usual dietaries of invalids, that every physician owes a debt of gratitude to Messrs. Brand for putting at his disposal so varied and so extensive a menu of wholesome and nutritious specialities. The invalid's dietary need no longer consist of milk and beef tea, beef tea and milk, for without having to depend on the vagaries or caprices of the ordinary household cook, thanks to this firm, the medical attendant may make his daily selection from some of the following: Mutton broth, veal broth, chicken broth, game broth, turtle broth, mutton jelly, chicken jelly, beef tea jelly, calf's foot, ivory, lemon, or orange jelly, and a whole host of other specialities which are to be found in Brand's catalogue, and all of which are first-class of their kind.

